# Inhibition of ABCC6 Transporter Modifies Cytoskeleton and Reduces Motility of HepG2 Cells via Purinergic Pathway

**DOI:** 10.3390/cells9061410

**Published:** 2020-06-05

**Authors:** Angela Ostuni, Monica Carmosino, Rocchina Miglionico, Vittorio Abruzzese, Fabio Martinelli, Daniela Russo, Ilaria Laurenzana, Agata Petillo, Faustino Bisaccia

**Affiliations:** 1 Department of Sciences, University of Basilicata, viale Ateneo Lucano 10, 85100 Potenza, Italy; monica.carmosino@unibas.it (M.C.); rocchina.miglionico@virgilio.it (R.M.); v.abruzz@hotmail.it (V.A.); fabiomartinelli@alice.it (F.M.); daniela.russo@unibas.it (D.R.); agata.petillo@unibas.it (A.P.); 2 Laboratory of Preclinical and Translational Research, IRCCS-Referral Cancer Center of Basilicata (CROB), 85028 Rionero in Vulture, Italy; ilaria.laurenzana@crob.it

**Keywords:** ABCC6, HepG2, probenecid, purinergic pathway, ATP, cytoskeleton, cell motility

## Abstract

ABCC6, belonging to sub-family C of ATP-binding cassette transporter, is an ATP-dependent transporter mainly present in the basolateral plasma membrane of hepatic and kidney cells. Although the substrates transported are still uncertain, ABCC6 has been shown to promote ATP release. The extracellular ATP and its derivatives di- and mono-nucleotides and adenosine by acting on specific receptors activate the so-called purinergic pathway, which in turn controls relevant cellular functions such as cell immunity, inflammation, and cancer. Here, we analyzed the effect of *Abcc6* knockdown and probenecid-induced ABCC6 inhibition on cell cycle, cytoskeleton, and motility of HepG2 cells. Gene and protein expression were evaluated by quantitative Reverse Transcription PCR (RT-qPCR) and western blot, respectively. Cellular cycle analysis was evaluated by flow cytometry. Actin cytoskeleton dynamics was evaluated by laser confocal microscopy using fluorophore-conjugated phalloidin. Cell motility was analyzed by in vitro wound-healing migration assay. Cell migration is reduced both in *Abcc6* knockdown HepG2 cells and in probenecid treated HepG2 cells by interfering with the extracellular reserve of ATP. Therefore, ABCC6 could contribute to cytoskeleton rearrangements and cell motility through purinergic signaling. Altogether, our findings shed light on a new role of the ABCC6 transporter in HepG2 cells and suggest that its inhibitor/s could be considered potential anti-metastatic drugs.

## 1. Introduction

Extracellular ATP, but also adenosine, ADP, UTP, UDP, participate in the “purinergic signaling” pathway, a ubiquitous system of cell-to-cell communication that modulates several pathophysiological processes such as wound healing, cancer, neurodegeneration, and inflammation [1]. Four families of specific receptors (i.e., nucleotide P2X and P2Y receptors, adenosine P1 receptors, the adenine-selective P0 receptor and several ectonucleotidases) are essential components of this pathway. Nucleotides can be released from damaged cells, plasma membrane-derived microvesicles, membrane channels (connexins, pannexins, calcium homeostasis modulator channels, and P2 × 7 receptor) or specific ATP binding cassette (ABC) transporters [2,3].

The human *Abcc6* gene belongs to the ABCC family and codifies for the MRP6 protein, mainly expressed at the basolateral membrane of hepatocytes [4]. More than 300 mutations in *Abcc6* such as deletions, insertions, or substitutions, mostly occurring in the sequence encoding the nucleotide binding domains, are associated with the Pseudoxanthoma elasticum, an autosomal recessive disease characterized by a progressive ectopic calcification [5,6,7]. Studies aimed at understanding the molecular mechanisms underlying the observed phenotype have shown that ABCC6 contributes to the outflow of ATP from cells [8,9]. In the extracellular milieu, ATP is hydrolyzed by the ectonucleotidase ENPP1 to AMP and pyrophosphate (PPi), an inhibitor of ectopic mineralization. The ecto-5′-nucleotidase CD73 is able to catalyze the conversion of extracellular AMP to adenosine and phosphate; it is the main source of extracellular adenosine in all tissues in which ATP is poorly present in extracellular fluids [10,11]. CD73 is considered a key regulator in some cancer processes such as drug resistance, tumor metastasis, and tumor angiogenesis [12,13], therefore is an excellent candidate for cancer therapy [14,15,16]. In previous studies, we have reported that knockdown of *Abcc6* in hepatocarcinoma cancer cells (HepG2), or the inhibition of its activity lead to the downregulation of *NT5E* gene, which codifies for the CD73 protein [17,18,19]. Taken together, these data suggest a close correlation between ABCC6 and CD73.

Different roles of ABCC6 in various cancer types have been reported; it has been given an irrelevant [20,21], important [22,23,24,25], diagnostic [26,27] or prognostic [28,29,30] role. Furthermore, ABCC6 could play a role in cancer cell biology as an ATP supplier of the purinergic pathway.

In the present study, we demonstrated that both *Abcc6* silencing and ABCC6 inhibition, by modulating the extracellular pool of ATP, lead to cytoskeletal rearrangement and reduction in cell motility in HepG2 cells. Moreover, we suggest that probenecid, a drug with uricosuric activity as well as an unspecific inhibitor of some transport proteins including ABCC6 [31,32,33], might be a potential anticancer drug.

## 2. Materials and Methods

### 2.1. Cell Culture and Treatments

Human hepatoblastoma cells (HepG2) and triple-negative human breast cancer poorly differentiated cells (MDA-MB-231) were maintained in Dulbecco’s modified Eagle’s medium (DMEM) containing 4.5 g/L glucose, supplemented with 10% fetal bovine serum (FBS), 2 mM l-glutamine, 100 U/mL penicillin, and 100 µg/mL streptomycin at 37 °C, in an atmosphere humidified with 5% of CO_2_. Probenecid and adenosine were dissolved in dimethyl sulfoxide (DMSO) at 30 mg/mL and 80 mg/mL, respectively. Based on our previous cytotoxicity assays, 250 μM probenecid was selected for the experiments [18]. The final concentration of DMSO did not exceed 0.25% *v*/*v*; control cells were treated at the same final percentage of DMSO (vehicle). ATP and adenosine 5’-(α,β-methylene)diphosphate (AOPCP) were dissolved in phosphate buffered saline at 27.5 mg/mL and 50 mg/mL, respectively. The stock solutions were then diluted with DMEM to the desired concentrations. All compounds were purchased from Sigma (Sigma, Saint Louis, MO, USA).

### 2.2. Generation of Stable Abcc6 Knockdown HepG2 Cells

The *Abcc6* knockdown HepG2 cell line was established using a Lentivirus shRNA knockdown vector system purchased from Cyagen Biosciences (Santa Clara, CA, USA) with EGFP as a reporter gene. Cell transfection was performed according to the manufacturer’s instructions. Cell seeding was performed at a density of 1.5 × 10^5^ cells in a 12-well plate. After 24 h, the cells were transfected with both Abcc6-shRNA and scrambled-shRNA as a negative control, at a suitable multiplicity of infection equal to 10. In order to stably harvest knockdown cells, HepG2 cells were selected with 2 μg/mL puromycin for 12 days. After selection, individual resistant clones were expanded in medium without puromycin and clones silenced between 70 and 80% were used for further experiments.

### 2.3. Real-Time Reverse Transcription Polymerase Chain Reaction (RT-qPCR)

HepG2 cells cultured in the presence of 250 µM probenecid or 0.25% DMSO (vehicle) for 48 h were harvested and total RNA was extracted using the Quick-RNA MiniPrep Kit (Zymo Research, Irvine, CA, USA), according to the manufacturer’s protocol. cDNA was synthesized using a High-Capacity cDNA Reverse Transcription Kit (Applied Biosystem, Waltham, MA, USA) in accordance with the manufacturer’s instructions. Real-time quantitative RT-PCR was performed with a 7500 Fast Real-Time PCR System (Applied Biosystems) using iTaq™ Universal-SYBR^®^ Green Supermix (Bio-Rad, Hercules, CA, USA). To confirm PCR specificity, the PCR products were subjected to a melting-curve analysis. The comparative threshold cycle method (∆∆Ct) was used to quantify relative amounts of product transcripts with β-actin as the endogenous reference. Primers were designed for spanning exon–exon junctions eliminating undesirable genomic DNA amplification (Table 1).

### 2.4. Western Blot Analysis

Cells were lysed in Radioimmunoprecipitation assay (RIPA) buffer (0.1% sodium dodecyl sulfate, 1% NP-40, and 0.5% sodium deoxycholate in PBS at pH 7.4) supplemented with a protease and phosphatase inhibitor cocktail (Roche, Penzberg, Germany). The proteins (100 μg) were loaded onto 8% sodium dodecyl sulfate–polyacrylamide gels electrophoresis and electrophoretically transferred to nitrocellulose membranes (Amersham Bioscience, Buckinghamshire, UK). The membranes were blocked in saturation buffer (with 5% nonfat milk in PBS with 0.05% Tween 20, PBST) for 2 h at room temperature and then probed overnight at 4 °C with specific primary antibodies: 1:400 anti-β-actin monoclonal (Sigma, Saint Louis, MO, USA); 1:50 MRP6 Monoclonal Antibody (M6II-31) (Invitrogen, Waltham, MA, USA); 1:500 anti-CD73 monoclonal (Invitrogen); and 1:1000 anti-Lamin A/C polyclonal (Cell Signaling, Danvers, MA, USA). Membrane was washed three times with PBST and then incubated at room temperature for 1 h with appropriated horseradish peroxidase-conjugated secondary antibody and signal visualized by ECL™ Western Blotting Detection Reagents (GE Healthcare, Chicago, IL, USA) or SuperSignal™ West Pico PLUS Chemiluminescent Substrate (Thermo Scientific, Waltham, MA, USA), using a Chemidoc™ XRS detection system equipped with Image Lab Software for image acquisition (BioRad). Densitometric analysis was performed by using GelAnalizer 2010 software (Istvan Lazar, www.gelanalyzer.com). Protein expression level in the control sample was taken as 100%. Each result was expressed as a percentage of the value of the control sample. Each test was repeated three times.

### 2.5. Extracellular ATP Bioluminescence Assay

Measurement of extracellular ATP in cell culture medium was performed through the ATP Bioluminescence Assay Kit CLS II (Roche), according to the manufacturer’s protocol. A total of 1.5 × 10^5^ HepG2 cells and 1 × 10^5^ MDA-MB-231 cells were seeded into each well of a 12 well plate and after 24 h, the culture medium was removed, cells were washed with PBS and complete DMEM medium without Phenol Red (D5030 Sigma-Aldrich, Saint Louis, MO, USA), in the presence and absence of probenecid 250 µM, was added. After 48 h, 100 µL of the culture medium was gently collected in ice-cold sterile tubes, centrifugated at 1200 rpm for 5 min at 4 °C to precipitate cell debris, and ATP in the supernatant was measured after dilution to 1:10 in ATP free water and the addition of a luciferin/luciferase reagent. Luminescence was immediately determined through a Glomax luminometer (Promega, Madison, WI, USA) with an integration time of 0.5 s^−1^. ATP content was determined quantitatively by luminometry, and the ATP concentrations were normalized to an ATP standard curve. ATP levels were then normalized per micrograms of proteins.

### 2.6. Cell Cycle Analysis

Cell cycle analysis was performed as described by Laurenzana et al. [34]. Briefly, HepG2 cells (2 × 10^5^ cells/well) were seeded into 12-well plates and treated with 250 µM probenecid or 0.25% DMSO (vehicle) for 24 and 48 h. Cells were then harvested, washed once with ice-cold PBS, fixed in cold 70% ethanol, and stained with propidium iodide staining solution (50 μg/mL PI and 10 μg/mL RNase) for 30 min in the dark. Cells were analyzed for cell cycle distribution using Navios flow cytometer (Beckman Coulter, O’Callaghan’s Mills, Ireland) and ModFit LT Software (Verity Software House, Topsham, ME, USA).

### 2.7. Confocal Fluorescence Microscopy

HepG2 cells (1.5 × 10^5^) and MDA MB 231 cells (1 × 10^5^) were grown on coverslips and treated with 250 µM probenecid (in presence or absence of 500 µM ATP and 100 µM adenosine) or 50 µM AOPCP for 48 h. Cells were fixed in 4% PFA for 10 min at room temperature, washed three times with PBS, permeabilized with 0.5% *v*/*v* Triton X-100 in PBS for 5 min, washed three times with PBS, and blocked in saturation buffer (1% bovine serum albumin in PBS) for 20 min. Then, cells were incubated with Phalloidin Alexa Fluor 488 (ex./em.: 495/518 nm, Invitrogen™) diluted 1:1000 or Phalloidin Alexa Fluor 568 (ex./em.: 578/600 nm, Invitrogen™) diluted 1:500 in saturation buffer for 1 h, in the dark and after three washes with PBS. Where indicated, the nuclei were stained with 1.5 µM of propidium iodide (ex./em.: 535/617 nm, Invitrogen™) in PBS for 10 min. The images were obtained with a confocal fluorescence microscope (Leica TSC-SP2 HCX PL APO, ×63/1.32–0.60 oil objective) and acquired using the Leica Confocal Software W (Leica Microsystem, Wetzlar, Germany).

### 2.8. Migration Assay

Cell migration rate was evaluated by the in vitro wound-healing migration assay. To assess the effect of probenecid, HepG2 cells (1 × 10^6^) were seeded in each 35-mm cell culture dish and cultured in DMEM containing 10% FBS to promote a nearly confluent cell monolayer. Twenty-four hours after seeding, cells were treated with either 250 µM probenecid or 0.25% DMSO for 36 h in DMEM containing 10% FBS. Then, a linear wound was generated in the cellular monolayer with a sterile 10 μL plastic pipette tip. Any cellular debris was removed by washing with PBS and replacing with 2 mL of DMEM with 1% FBS still containing 250 µM probenecid or 0.25% DMSO and placed in the BioStation IM incubator, version 2 (Nikon, Tokyo, Japan) for 12 h. Please note that cells were treated overall for 48 h with 250 µM probenecid or 0.25% DMSO. Time-lapse images were obtained every hour for 12 h and further analyzed by using computing software (ImageJ 1.46, ImageJ 1.46, U. S. National Institutes of Health, Bethesda, MD, USA). Migration rate was reported as µm/h.

Abcc6-shRNA HepG2 cells (1 × 10^6^) were cultured in DMEM containing 10% FBS to promote a nearly confluent cell monolayer. Twenty-four hours after seeding cells, a linear wound was generated in the cellular monolayer with a sterile 10 μL plastic pipette tip. Any cellular debris was removed by washing with PBS and replacing with 2 mL of DMEM with 1% FBS and placed in the BioStation IM incubator, version 2 (Nikon) for 12 h. Images were analyzed as indicated above.

### 2.9. Statistical Analysis

All of the assays were performed at least three times independently. Statistical analyses were performed using the Student’s *t*-test or one-way Analysis of variance (ANOVA). Where indicated, data are presented as the means ± standard error (ES) by GraphPad Prism software (GraphPad Software, San Diego, CA, USA). * *p* < 0.05, ** *p* < 0.01, and *** *p* < 0.001 were considered to be statistically significant.

## 3. Results

### 3.1. Probenecid Affects the Extracellular ATP Released by HepG2 Cells

**Figure 1:**
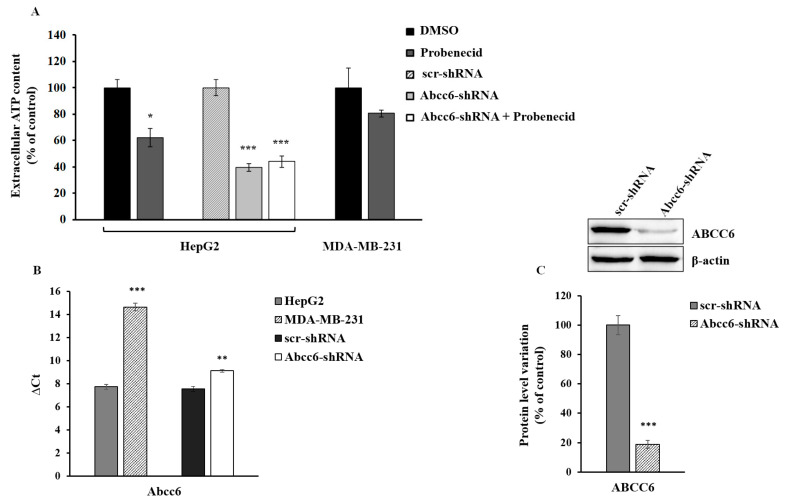
Extracellular ATP content. (**A**) HepG2 and MDA-MB-231 cells were treated with 250 μM probenecid or 0.25% DMSO for 48 h. Results are expressed as the mean ± standard error (ES) of three different experiments. * *p* < 0.05 Probenecid vs. DMSO, *** *p* < 0.001 Abcc6-shRNA and Abcc6-shRNA + Probenecid vs. scr-shRNA (**B**) *Abcc6* gene expression in HepG2, Abcc6-shRNA HepG2 and MDA-MB-231 cells. Gene expression was normalized to β-actin mRNA levels. *** *p* < 0.001 MDA-MB-231 cells vs. HepG2 cells, ** *p* < 0.01 Abcc6-shRNA HepG2 cells vs. scrambled HepG2 cells; (**C**) Representative western blot of Abcc6-shRNA HepG2 cells. Densitometric analysis of the immunoreactive bands performed in five independent experiments. The protein levels were normalized with β-actin content. Data were normalized to scrambled cells set to 100%. Data are shown as the mean ± standard error (SE) *** *p* < 0.001 Abcc6-shRNA HepG2 cells vs. scr-shRNA HepG2 cells.

There are multiple systems through which ATP can leave cells including ABCC6 [8,9]. It has also been shown that probenecid inhibits both the expression [18] and the activity of the ABCC6 transporter [33]. In order to evaluate the contribution of ABCC6 to the outflow of ATP from HepG2, we measured the amount of ATP released from the silenced and probenecid-treated cells in comparison with MDA-MB-231 cells, which, compared to HepG2, have a lower expression of *Abcc6* (Figure 1). When HepG2 cells were treated with 250 μM probenecid for 48 h, we observed a decrease in the amount of extracellular ATP by about 40% (Figure 1A). *Abcc6* silencing reduced the ATP levels by about 60% compared to those of the control cells (scr-shRNA) and did not decrease further with the addition of probenecid, thus suggesting a significant contribution of the ABCC6 transporter to the extracellular ATP pool. On the contrary, in MDA-MB-231 cells, in which the expression of *Abcc6* was from low to negligible compared to HepG2 cells (Figure 1B), probenecid did not affect the extracellular ATP content, thus suggesting that the ABCC6 transporter is scarcely involved in the extrusion of ATP from these cancer cells.

### 3.2. Probenecid Affects the Expression of Some Genes in HepG2 Cells

**Figure 2:**
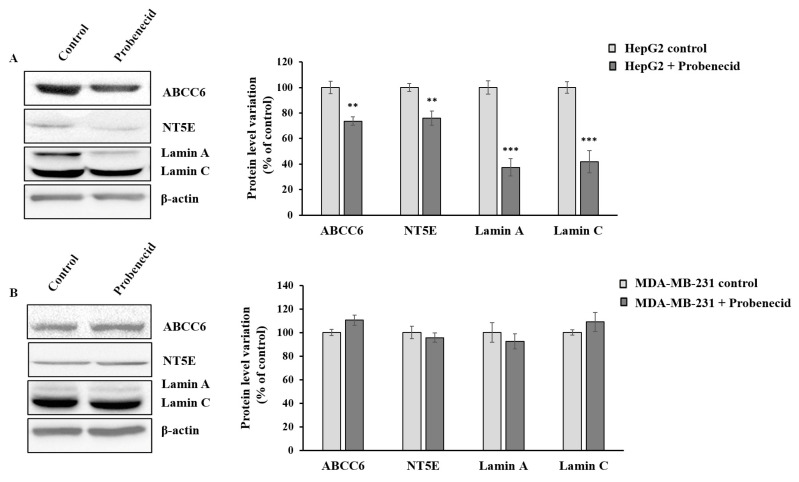
Effect of probenecid on ABCC6, NT5E, and Lamin A/C expression. (**A**) Representative western blot of HepG2 cells and (**B**) MDA-MB-231 cells treated with 250 µM probenecid or 0.25% DMSO (control) for 48 h. Densitometric analysis of the immunoreactive bands performed in three independent experiments. The protein levels were normalized with β-actin content. Data were normalized to control cells set to 100%. Data are shown as the mean ± standard error (SE) ** *p* < 0.01, *** *p*< 0.001 of HepG2 + Probenecid vs. HepG2 control.

We previously demonstrated that the downregulation of *Abcc6* disregulates the expression of CD73 and Lamin A/C proteins. To verify that this effect is specific for cells expressing appreciable levels of ABCC6 transporter, we tested the effect of probenecid on the expression level of CD73 and Lamin A/C in both HepG2 (Figure 2A) and MDA-MB-231 cells (Figure 2B). Interestingly, unlike for that observed in the HepG2 cells, the expression levels of these genes did not change in MDA-MB-231 cells upon probenecid treatment, confirming that the effects of probenecid on gene expression in HepG2 cells is selective and could be largely mediated by mechanism/s involving the ABCC6 transporter.

### 3.3. Probenecid Does Not Affect HepG2 Cell Cycle

**Figure 3:**
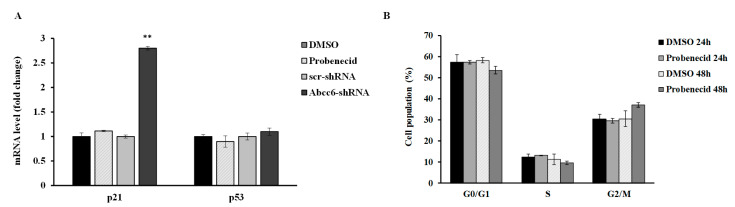
(**A**) mRNA expression levels of p21 and p53 genes in HepG2 cells treated with 250 µM probenecid for 48 h and in *Abcc6*-knockdown HepG2 cells. Data are shown as fold increase compared with DMSO-treated cells calibrator for HepG2 probenecid treated cells (Probenecid) or scrambled HepG2 cells (scr-shRNA) calibrator for Abcc6-shRNA HepG2 cells. (**B**) Flow cytometry analysis of cell cycle phase distribution of HepG2 cells treated with 250 µM probenecid for 24 and 48 h. Data are expressed as means ± standard error (SE) of three replicates from three independent experiments. ** *p* < 0.01 Abcc6-shRNA vs. scr-shRNA.

We have previously shown cell cycle alteration with a senescence-like phenotype in *Abcc6*-silenced HepG2 cells [19]. HepG2 cells treated with probenecid showed neither alteration of cyclin-dependent kinase inhibitor p21/WAF1 and Tumor Protein p53 (p53) expression, nor alteration of the cell cycle (Figure 3).

### 3.4. Probenecid Induces Cytoskeletal Rearrangement of HepG2 Cells

Considering that Lamin is among the structural components of the cytoskeleton and its expression changed in probenecid treated HepG2 cells, we analyzed the effect of probenecid on cytoskeleton arrangement. Actin filaments (also called F-actin) are the main component of the cytoskeleton and are involved in cellular movements [35]. The initial step in cell migration is the protrusion of the leading cell membrane created by branched, dendritic arrays, the filopodia.

**Figure 4:**
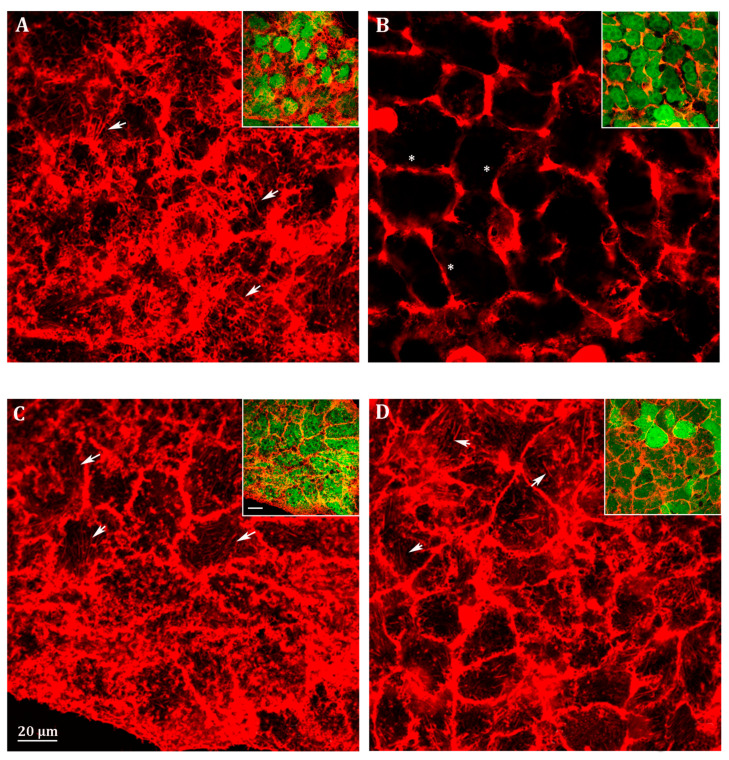
Representative confocal image of (**A**) scrambled HepG2 cells; (**B**) Abcc6-shRNA HepG2 cells; (**C**) Abcc6-shRNA HepG2 cells treated with 500 µM ATP; (**D**) Abcc6-shRNA HepG2 cells treated with 100 µM adenosine. F-actin was stained with Phalloidin Alexa Fluor 568. In the insets, superposition of cytoskeleton (red) and EGFP (green) to monitor the infection efficiency. The scale bar in the enlarged figure: 40 µm.

We analyzed filopodia in *Abcc6* knockdown HepG2 cells by confocal fluorescence microscopy (Figure 4). The efficiency of stable silencing in each experimental condition is appreciable in the relative insets in Figure 4. In fact, since the viral vectors used for *Abcc6* silencing incorporate the sequence of EGFP as a gene reporter, cells infected with either the scrambled shRNA or the Abcc6-shRNA showed a green-labeled cytoplasm when visualized with filters for EGFP (Figure 4, insets). Scrambled HepG2 cells exhibited many filopodia, which extended in all directions in the cells (Figure 4A, arrows). Instead, in Abcc6-shRNA HepG2 cells, filopodia were almost completely absent (Figure 4B, stars); the addition in *Abcc6* silenced cells of either adenosine (Figure 4C, arrows) or ATP (Figure 4D, arrows) restored the normal architecture of filopodia.

**Figure 5:**
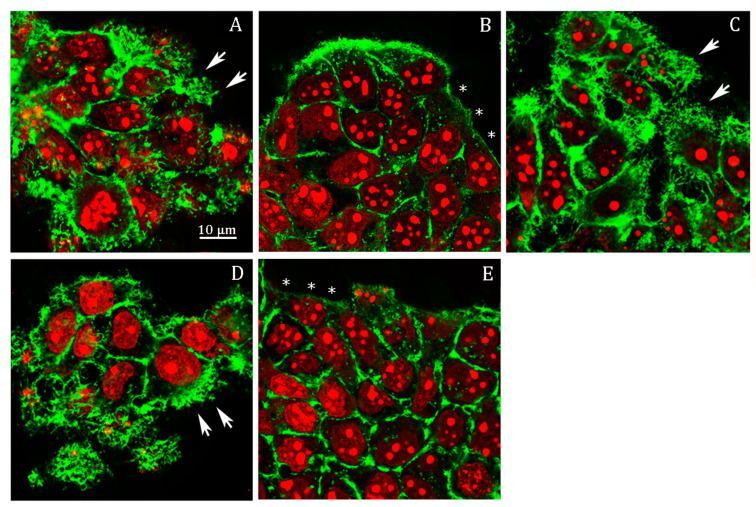
Representative confocal image of HepG2 cells treated with (**A**) 0.25% DMSO (vehicle) for 48 h; (**B**) 250 µM probenecid for 48 h; (**C**) 250 µM probenecid and 500 µM ATP for 48 h; (**D**) 250 µM probenecid and 100 µM adenosine for 48 h; (**E**) 50 µM AOPCP for 48 h. F-actin and nuclei were stained with Phalloidin Alexa Fluor 488 and propidium iodide, respectively.

In probenecid-treated HepG2 cells, we observed an almost complete lack of filopodia, or, if present, they were very short (Figure 5B, stars) compared to the control cells (Figure 5A, arrows); as observed in the Abcc6-shRNA cells, the addition of ATP (Figure 5C, arrows) or adenosine (Figure 5D, arrows) restored the normal architecture of filipodia.

Previously, we showed that ATP as well as adenosine reverted the effect of probenecid on the downregulation of the protein level of CD73, restoring its protein level to that of the control conditions and even above [18]. The finding that adenosine alone mimicked the effect of ATP in this experiment suggests that the ability of ATP to revert the effect of probenecid on cytoskeleton architecture is likely dependent on its ability to be finally transformed in adenosine by CD73. To verify this possibility, we analyzed filipodia organization upon inhibition of CD73 with adenosine 5’-(α,β-methylene)diphosphate (AOPCP), a non-hydrolysable ADP analog, which binds to the active site and inhibits the catalytic activity of CD73 (Figure 5E). AOPCP is able to recapitulate the effect of probenecid with almost absent filipodia (Figure 5E, arrows), thus indicating that the lack of extracellular adenosine, derived from extracellular ATP, causes the filipodia rearrangement.

**Figure 6:**
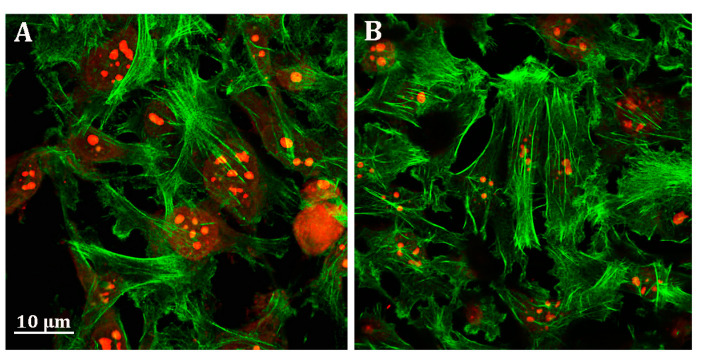
Representative confocal image of MDA-MB-231 cells treated with (**A**) 0.25% DMSO (vehicle) and (**B**) 250 µM probenecid for 48 h. F-actin and nuclei were stained with Phalloidin Alexa Fluor 488 and propidium iodide, respectively.

Unlike HepG2 cells, no effect of probenecid was observed on the MDA-MB-231 cell cytoskeleton (Figure 6A,B).

### 3.5. Probenecid Affects HepG2 Cell Migration

**Figure 7:**
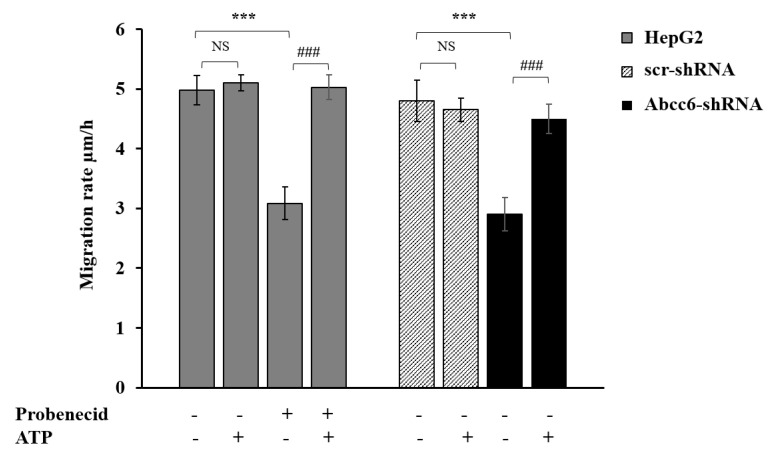
Effect of probenecid and *Abcc6* silencing on the migration rate of HepG2 cells. Cells were treated with 250 μM probenecid for 48 h (gray plain bars, Probenecid+). DMSO-treated cells were used as the control (gray plain bars, Probenecid−). A total of 500 µM ATP was added to either the control cells (gray plain bars, Probenecid−, ATP+) or to probenecid-treated cells (gray plain bars, Probenecid+, ATP+). HepG2 cells were transduced with scrambled shRNA (grey-texturized bars, scr-shRNA) or with specific Abcc6-shRNA (black bars, Abcc6-shRNA). A sample of 500 µM ATP was added to either control cells (grey-texturized bars, scr-shRNA, ATP+) or to *Abcc6* silenced cells (black bars, Abcc6-shRNA, ATP+). Data are expressed as mean ± standard error (SE) of three replicates from three independent experiments. Data were analyzed by one-way ANOVA followed by Dunnett’s post hoc test using GraphPad Prism 7 software, *** *p* < 0.001 probenecid treated cells (gray plain bars, Probenecid+) vs. control cells (gray plain bars, Probenecid−) in the absence of ATP (ATP−); Abcc6-shRNA (black bars, Abcc6-shRNA) vs. scr-shRNA (grey-texturized bars, scr-shRNA) in the absence of ATP (ATP−). ^###^ *p* < 0.001 probenecid + ATP treated HepG2 cells (gray plain bars, Probenecid+, ATP+) vs. probenecid treated HepG2 cells (gray plain bars, Probenecid+, ATP−); Abcc6-shRNA cells+ ATP (black bars, Abcc6-shRNA, ATP+) vs. Abcc6-shRNA cells (black bars, Abcc6-shRNA, ATP−). NS, not significant.

Cell migration is regulated and supported by both specific soluble molecules and different intracellular pathways including those involved in cytoskeletal rearrangement [36]. We indeed evaluated cell migration upon *Abcc6* silencing and ABCC6 inhibition by the scratch-wound assay. *Abcc6* knockdown cells (Abcc6-shRNA) showed a significant inhibition of about 40% of migration rate compared to control cells (scr-shRNA) (Figure 7). Probenecid elicited the same effect of *Abcc6* silencing in HepG2 cells on cell migration rate (Figure 7, Probenecid −/+). The addition of ATP completely reverted the effect of both *Abcc6* knockdown and probenecid treatment in HepG2 cells (Figure 7, +ATP). In the presence of ATP, indeed, the cell migration rate was restored to that of resting condition, specifically to 4.8 ± 0.35 μm/h and 5.0 ± 0.21 μm/h in the silenced and in probenecid treated cells, respectively. Overall, these results indicate a significant role of ABCC6 on HepG2 cell migration.

## 4. Discussion

The human *Abcc6* gene encodes for a protein mainly expressed in the basolateral membrane of the hepatocytes. In order to understand the possible physiological substrate/s transported and then the molecular mechanisms underlying the clinical phenotype of PXE, a number of studies have been conducted to clarify the structure and function of some domains of ABCC6 [37,38,39,40,41,42]. To date, we know that ABCC6 contributes to the outflow of ATP from cells [8,9]. ATP contributes to supplying the extracellular space of the anti-mineralization factor PPi, however, in a biological context such as that of tumors, we suggest that the role of ABCC6 can be as an ATP supplier in the purinergic pathway. Both nucleotides and nucleosides are important modulators of tumor biology and the purinergic system contributes with different mechanisms to the regulation of cancer growth and dissemination [43,44]. In the present study, we analyzed the effect of *Abcc6* knockdown and probenecid-induced ABCC6 inhibition on the extracellular ATP availability, and consequently on cell migration, an important cellular process correlated to cancer.

We found that either *Abcc6* silencing or ABCC6 inhibition with probenecid induced a comparable decrease in the extracellular ATP content in HepG2 cells (Figure 1). Moreover, when *Abcc6* silenced cells were treated with probenecid, no additive effects were observed, thus suggesting that, at least in HepG2 cells, probenecid inhibits the outflow of ATP from ABCC6 or ABCC6-related transport systems [45].

The hypothesis that the availability of ATP may be responsible for the cytoskeleton rearrangement and the consequent inhibition of cell migration has also been demonstrated. The cytoskeleton architecture in *Abcc6* knockdown HepG2 cells is drastically modified (Figure 4A,B) as well as cell motility (Figure 7). Both conditions can be mimicked by the addition of probenecid to HepG2 cells and restored by the addition of ATP (Figure 4D, Figure 5, and Figure 7): as long as the outflow of ATP from the cells is not inhibited with probenecid, its amount is sufficient for the formation of filopodia and for motility.

Since we have observed that both *Abcc6* silencing [19] and probenecid treatment induced the down regulation of Lamin A/C expression (Figure 2A), we suggest that the lower levels of Lamin A/C can contribute to the rearrangement of the cytoskeleton and consequently to changes in cell migration. Accordingly, overexpression of Lamin A/C correlates with high degrees of malignance of some tumors due to its ability to promote migration and invasion, downregulation, or absence of Lamin A/C is associated with the low to absent cell motility [46].

In HepG2 cells, we have already observed a correlation between a decrease in the expression levels of CD73 upon *Abcc6* silencing [17] or probenecid treatment [18]. CD73 is the main source of extracellular adenosine in all tissues and is a key regulator in some tumor processes such as invasion, migration, and metastasis [12,13]. In this study, we found that the inhibition of CD73 activity by AOPCP (Figure 5E) mimicked the effect of probenecid in the filipodia retraction; however, the addition of adenosine together with probenecid preserved the filipodia architecture, suggesting that ABCC6 could modulate the extracellular adenosine availability with a combined action on the rate of ATP efflux and, at same time, on the expression levels of the CD73 enzyme. As CD73 also functions as a membrane receptor for extracellular matrix proteins [47], it is not excluded that probenecid, downregulating its expression may affect adhesion, migration, and cellular invasion.

However, despite all that we have discussed so far, not all effects observed following *Abcc6* silencing were mimicked by treatment with probenecid. Unlike what was previously observed in *Abcc6* knockdown HepG2 cells [14], probenecid did not block cell cycle and cellular senescence, probably because it has a lesser impact on cellular metabolism, compared to what happens in response to gene silencing.

## 5. Conclusions

ABCC6 could contribute to cytoskeleton rearrangements and cell motility through purinergic signaling by contributing to the extracellular reserve of ATP. Pharmacological inhibition of ABCC6 by probenecid roughly mimics the effects of genetic silencing by regulating cytoskeleton rearrangement and cell motility, thus identifying ABCC6 as a potential therapeutic target for anti-metastatic treatment.

## Figures and Tables

**Table 1:** List of primers used in this study.

Gene	Accession Number	Forward Primer	Reverse Primer
β-actin	NM 001101.3	5′-CCTGGCACCCAGCACAAT-3′	5′-GCCGATCCACACGGAGTACT-3′
Abcc6	NM_001171.5	5′-AAGGAACCACCATCAGGAGGAG-3′	5′-ACCAGCGACACAGAGAAGAGG3′
p21	NM_000389	5′-CTGTCTTGTACCCTTGTGCCT-3′	5′-CGTTTGGAGTGGTAGAAATCTGTC-3′
p53	NM_001276760.1	5′-TGAATGAGGCCTTGGAACTC-3′	5′-ACTTCAGGTGGCTGGAGTG-3′
